# Lymphatic Obstruction and Edema in Neonate due to Left Subclavian Central Venous Catheter

**DOI:** 10.1155/crpe/2811167

**Published:** 2025-06-17

**Authors:** Klaas Koop, Dominique Valérie Clarence de Jel, Joppe Nijman, Barbara Peels, Ellis Peters

**Affiliations:** ^1^Department of Pediatrics, Wilhemina Children's Hospital, University Medical Center Utrecht, Utrecht, the Netherlands; ^2^Pediatric Intensive Care Unit, Wilhemina Children's Hospital, University Medical Center Utrecht, Utrecht, the Netherlands; ^3^Department of Neonatology, Leiden University Medical Center, Leiden, the Netherlands

**Keywords:** central venous catheter, lymphatics, neonate

## Abstract

There are several causes of generalized edema in sick neonates. We describe two newborns that developed progressive and treatment-resistant generalized edema. We suggest this is due to impaired lymphatic flow from the thoracic duct as a result of a central venous catheter in the left subclavian vein.

## 1. Introduction

Generalized edema is frequently seen in critically ill children. Causes of edema include increased capillary permeability (“capillary leak”), inability to excrete fluids (e.g., renal failure), and changes in hydrostatic and oncotic pressures that cause shifts of fluids to the interstitial compartment (e.g. hypoalbuminemia).

Here, we describe two neonates who developed severe generalized edema in the course of their neonatal intensive care unit (NICU) admission. We could not identify any of the abovementioned causes of edema. Treatment with fluid restriction and diuretics had no effect. Both children showed a swift resolution of edema after the removal of an intravenous catheter from the left subclavian vein. We suggest that obstruction of lymphatics at the level of the thoracic duct by subclavian vein catheters may cause generalized edema.

## 2. Case reports

**Figure 1:**
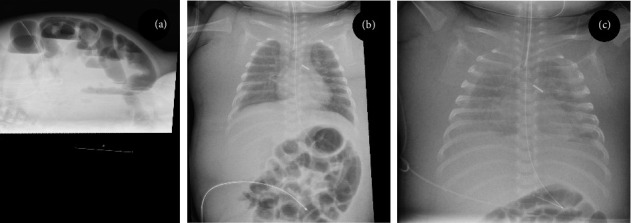
Abdominal X-ray of Patient A showing signs of bowel obstruction (a). On Day 8 after the operation, there is remarkable subcutaneous edema. Note the catheter in the left subclavian vein. Also, note the absence of edema in bowel loops (b). Despite intensive diuretic treatment edema persisted, and eventually pleural effusion developed (c).

Patient A was a two-month-old boy who was admitted to the NICU with abdominal distension. The patient was born at 27 weeks gestational age, birthweight 1215 g. In his early neonatal period, he developed infant respiratory distress syndrome for which he needed mechanical ventilation, he had a persistent ductus arteriosus that was ligated surgically, and he had developed necrotizing enterocolitis that had been treated conservatively. At the age of 2 months, he was readmitted to the NICU because of obstructive ileus (Figure 1(a)). On laparotomy, stenosis of a segment of the colon was found, probably resulting from the necrotizing enterocolitis. The stenotic segment was resected and an end-to-end anastomosis was made. The boy was given a left subclavian central venous catheter for parenteral nutrition.

After the operation, he developed severe sepsis and needed circulatory support with repeated crystalloid boluses for correction of hypotension. This not only stabilized the patient but also resulted in considerable generalized edema (Figure 1(b)). We considered this to be the result of a capillary leak during sepsis and expected the edema to subside once the boy's general situation improved. When after a few days the inflammation subsided, we were surprised to find that the generalized edema persisted and even worsened. The boy had no signs of infection and had normal hemodynamic parameters, cardiac evaluation, renal function, and renal ultrasound. He had hypoalbuminemia, for which we gave albumin infusions. Plasma sodium and potassium levels were normal. We started treatment with diuretics (eventually including furosemide, hydrochlorothiazide, spironolactone, and bumetanide), but this failed to increase urine production. Strikingly, despite the diuretic treatment, renal sodium excretion was relatively low (< 20 mmol/L). Over the course of 2 weeks, the edema worsened and the boy eventually developed bilateral pleural effusions (Figure 1(c)). On thoracentesis, this proved to be chylus. After drainage of the chylothorax, the generalized edema subsided. We suspected that the central venous catheter in the left subclavian vein blocked the thoracic duct, thereby impairing lymphatic flow and causing a chylothorax. On ultrasound, we found a thrombus surrounding the catheter, extending from the left subclavian vein into the brachiocephalic and jugular vein. After the removal of the central venous catheter and initiation of low molecular weight heparin therapy, the boy made a complete recovery.

Patient B was a two-month-old girl who was admitted to the NICU. She was born at the gestational age of 27 weeks with a birthweight of 670 g. In the first week of her life, she had developed a severe necrotizing enterocolitis. During laparotomy, part of her colon had to be resected and she was given an ileostomy. She had recovered from these initial problems and was now readmitted for the repair of the ileostomy.

**Figure 2:**
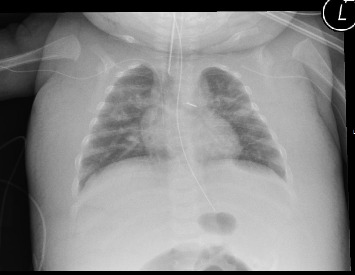
Chest X-ray of Patient B, showing remarkable subcutaneous edema and the presence of a central venous catheter in the left subclavian vein. The catheter on the left was removed after this X-ray confirmed the correct position of the right subclavian vein catheter.

After the laparotomy, she developed sepsis and generalized edema (Figure 2). The generalized edema persisted after the sepsis had subsided. She had normal cardiac and renal function. Treatment with diuretics had no effect. We therefore suspected that infection of the central venous catheter positioned in the left subclavian vein might be the cause of the persistent edema. After the replacement of the catheter, her condition improved and the edema subsided. Earlier in the course of the disease, she had developed thrombosis of the inferior vena cava, and thrombosis of the large thoracic vessels seemed likely. Unfortunately, we have not performed an ultrasound of the thoracic veins. The girl eventually recovered and is currently in good condition.

## 3. Discussion

We describe two neonates with unexplained refractory generalized edema and, in one patient, chylothorax. Both patients improved after removal of the central venous catheter from the left subclavian vein. We hypothesize that the generalized edema was caused by the impairment of lymphatic flow at the level of the thoracic duct.

The formation of interstitial fluid is determined by vascular permeability and the balance of hydrostatic and oncotic pressure in plasma and interstitial fluid. A surplus of interstitial fluid is transported back to the blood via lymphatic vessels. Generalized edema develops when there is an increase in (1) vascular permeability, or a relative increase in (2) plasma hydrostatic or (3) interstitial oncotic pressure. In critically ill patients, a combination of these factors is often present. In our patients, vascular permeability was probably increased as a result of sepsis, and infusion of large amounts of crystalloids resulted in increased plasma hydrostatic and decreased plasma oncotic pressure. However, when these initial events were over, both patients had persistent edema. We suggest that this is related to obstruction of the flow of lymphatic fluid via the thoracic duct.

Several mechanisms could explain the effect of lymphatic obstruction on edema. The edema might be purely “mechanical,” with the obstructed lymphatic duct leading to an increased intralymphatic pressure that prohibits the return of surplus interstitial fluid to the circulation. Indeed, increased intralymphatic pressure has been related to complications such as chylothorax [1], as was the case in Patient A. Central venous catheters and increased central venous pressure have been linked to chylothorax [1–6], and generalized edema has also been described in these patients [7].

Alternatively, but at this moment not supported by literature or experimental evidence, we hypothesize that the appearance and resolution of edema is a regulated process and that a blocked lymph flow perturbs this regulation. As can be seen from the abdominal X-ray (Figures 1(b) and 1(c)), “generalized edema” is in fact limited to the skin. This suggests that the accumulation of fluid in the skin, but not other organs, might be regulated. The edema seen in critical illness could thereby be an augmented version of a physiological function of the skin as “fluid reservoir.” Other physiologic processes in the skin, including salt retention, are under active lymphatic control [8, 9]. We suggest that lymphatics might also have a role in the regulation of the fluid retention in the skin. Currently, it is unknown whether such regulation exists, but it is tempting to speculate about edema as an active physiologic process spun out of control, rather than a passive process.

In conclusion, chylothorax and edema may be complications of central venous catheters in newborns. We suggest that, if possible, central venous catheters in the left subclavian vein should be avoided in small newborns to prevent the obstruction of the lymphatic system. In patients with edema or chylothorax, ultrasound to detect possible catheter-related thrombosis should be performed promptly.

## Data Availability

The data that support the findings of this study are available on request from the corresponding author. The data are not publicly available due to privacy or ethical restrictions.
